# The emerging role of miRNAs in biological aging and age-related diseases

**DOI:** 10.1016/j.ncrna.2025.05.002

**Published:** 2025-05-05

**Authors:** Rawad Turko, Amro Hajja, Ahmad M. Magableh, Mohammed H. Omer, Areez Shafqat, Mohammad Imran Khan, Ahmed Yaqinuddin

**Affiliations:** a College of Medicine, Alfaisal University, Riyadh, 11533, Saudi Arabia; b School of Medicine, Cardiff University, Wales, United Kingdom; c Research Centre, King Faisal Specialist Hospital and Research Centre, Jeddah, 21589, Saudi Arabia

**Keywords:** microRNAs, miRNAs, Biomarkers, Aging, Ageing, Neurodegeneration, Cancer

## Abstract

Ageing is a complex biological process characterised by the accumulation of molecular and cellular damage leading to functional decline and an increased risk of chronic disease and geriatric syndromes. Despite data showing that lifestyle modifications, such as caloric restriction and exercise, can lead to healthy ageing and greatly reduce the incidence of chronic disorders, no medical therapies exist to delay or prevent these conditions effectively. We also lack tools to effectively track the ageing process in a manner that predicts an individual's risk of chronic disease and assess response to lifestyle or medical interventions. This review explores the emerging role of microRNAs (miRNAs), which are small non-coding RNAs that regulate gene expression, as a unifying mechanism underlying the biology of ageing and age-related conditions, including cardiovascular and neurodegenerative diseases, metabolic syndromes, musculoskeletal disorders such as sarcopenia, osteoarthritis and osteoporosis, and various cancers. We also examine the interactions of miRNAs with various hallmarks of ageing, such as DNA damage, cellular senescence, and mitochondrial dysfunction. We then explore the challenges of translating miRNA-based approaches from preclinical promise to clinical utility, emphasizing the need for trial-level validation to correlate miRNA profiles with clinically meaningful, patient-centred endpoints. By consolidating these findings, this article puts miRNAs forward as a pivotal mechanism in geroscience, offering a novel framework to mitigate ageing-related multimorbidity and bridge the gap between lifespan and healthspan.

## 1 Introduction

Ageing can be defined as “the result of accumulation of cellular and molecular damage, leading to functional decline, chronic disease, and mortality” [1]. Several theories of ageing have emerged, but there is much debate and no consensus about which one is the most robust [2]. What is known is that unprecedented increases in life expectancy over the past century have led to an expanding geriatric population spending an increasing fraction of their life with age-related conditions. These conditions include metabolic, cardiovascular, neurodegenerative and musculoskeletal disorders and geriatric syndromes (e.g., frailty, mild cognitive impairment, incontinence, falls, loss of mobility). These diseases collectively constitute the leading causes of morbidity, mortality, and health expenditures worldwide, especially in the developed world, where life expectancy is highest [3]. Thus, although human lifespan (i.e., longevity) has increased, “healthspan”, which is the number of years an individual spends free from chronic disease, has decreased [3]. Hence, research into the mechanisms of ageing and lifestyle or medical interventions to effectively delay ageing and its associated morbidity carries significant public health and economic implications.

### 1.1 The emergence of geroscience

Ageing is universal, even affecting unicellular organisms. However, its rate varies greatly. This implies that different species, and even different members of the same species, have varying degrees of susceptibility to the biological processes governing ageing. Comparative biology has demonstrated that remarkably long-lived species, such as the naked mole rate and hydra, have delayed the onset of age-related disorders and appear less sensitive to the biological mechanisms of ageing [4]. These and similar findings have sparked great interest among researchers in understanding the biological mechanisms driving ageing in the hope of eventually understanding the mechanistic underpinnings promoting healthy ageing trajectories.

Leveraging this plasticity of ageing to increase human healthspan through lifestyle and pharmacologic interventions has been the subject of numerous hypotheses, such as the ‘compression of morbidity’ hypothesis coined by James Fries [5,6]. In recent years, the Geroscience Hypothesis has gained traction as a framework for understanding and addressing age-related diseases [7]. It posits that the biological processes driving ageing are the major risk factors for most chronic diseases. Thus, rather than treating each of these conditions in isolation, Geroscience proposes targeting the shared fundamental ageing processes, such as macromolecular dysfunction, inflammation, cellular senescence, and stem/progenitor cell exhaustion, to delay or prevent the onset of multiple diseases simultaneously [7]. In recent years, the focus of Geroscience has shifted to extending healthspan rather than just lifespan (quality over quantity of life) [8]. By focusing on modifiable aspects of biological ageing, Geroscience represents a paradigm shift from viewing ageing as a non-modifiable risk factor to one amenable to lifestyle and pharmacological interventions, thereby offering a framework for reducing the collective burden of age-related diseases in terms of morbidity, mortality, and health expenditures [9,10].

From purely a mechanistic standpoint, perhaps the most popular framework for understanding ageing is the “*Biological Hallmarks of Ageing”*, coined by Lopez-Otin and colleagues in 2013. They enumerated nine core biological mechanisms of ageing: genomic instability, telomere attrition, epigenetic alterations, loss of proteostasis, cellular senescence, mitochondrial dysfunction, deregulated nutrient sensing, stem cell exhaustion, and altered intercellular communication [11]. This list was expanded a decade later to include disabled macroautophagy, chronic inflammation (i.e., inflammageing), and gut microbiome dysbiosis [12]. The authors used pre-defined criteria that a process must fulfil for it to be considered a hallmark of ageing: it must accumulate with the natural ageing process, its acceleration in animal models must accelerate the onset of age-related morbidities, and its genetic and pharmacologic attenuation must alleviate these ailments.

Nonetheless, the most important clinical question of whether targeting these hallmarks may prolong human healthspan remains investigational.

### 1.2 Epigenetic alterations as hallmarks of ageing

Epigenetic alterations are a primary hallmark of ageing that partly initiate the processes of progressive molecular and cellular damage. Epigenetic alterations include age-related changes in DNA methylation, histone post-translational modifications (PTMs), and non-coding RNA (ncRNAs), including microRNAs (miRNAs), long non-coding RNAs, and circular RNAs (circRNAs). These mechanisms collectively regulate the transcription and translation of genes into protein (i.e., gene expression), thereby shaping almost every aspect of cellular physiology.

Age-related changes to the epigenome were first described in animals (e.g., salmon and mice) and human fibroblasts, showing that DNA methylation changes and chromatin remodelling are related to organismal and cell ageing [[13], [14], [15], [16]]. Subsequent studies revealed that specific epigenetic alterations, such as the differential (de-)methylation/acetylation of particular histone residues, are associated with lifespan, healthspan, and disease. Likely, the plasticity of lifespan/healthspan across similarly chronologically aged humans and animals may, at least partly, reflect a biological driver that is sensitive to myriad intrinsic and extrinsic factors (e.g., genetic makeup, chronological age, lifestyle and environmental factors, disease states, and medical therapies). In this respect, the epigenome is indeed remarkably dynamic.

**Fig. 1:**
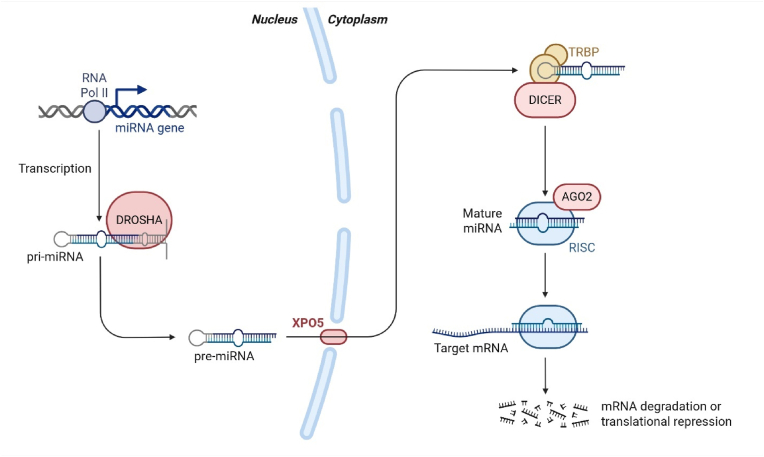
**miRNA synthesis and maturation.** DNA regions encoding miRNAs can be transcribed by RNA polymerase II, which initially forms a primary miRNA (pri-miRNA) cleaved by DROSHA to form pre-miRNA. This then translocates to the cytosol and undergoes cleavage by DICER to form mature miRNA. When bound to Argonaute (AGO) proteins, these molecules form the RISC, which can bind to target mRNAs and either facilitate their degradation or suppress their translation.

A large part of the human genome does not code for proteins. When transcribed, a large percentage of these genes produce ncRNAs. A significant proportion of these function as microRNAs, a class of small (∼17–25 nucleotides long) ncRNAs first was described in *Caenorhabditis elegans* (*C.* *elegans*) in 1993 [17]. The genes encoding miRNAs are transcribed into primary miRNA (pri-miRNA). The pri-miRNA undergoes cleavage processes in the nucleus (via Drosha-DGCR8 endonuclease to form pre-miRNA) and cytoplasm (via DICER) to form mature miRNAs that are incorporated into the RNA-induced silencing complex (RISC). When bound to their complementary mRNA, miRNAs either induce mRNA degradation or inhibit translation. Through this mechanism, miRNAs exert widespread control over various biological processes, including cell differentiation, proliferation, apoptosis, metabolism, and inflammation. We refer to other reviews on the biogenesis and maturation of miRNAs [18,19]. Fig. 1 illustrates the basic process of miRNA synthesis.

Alterations in the miRNA profile of different cells, tissues, organs, and body fluid compartments are part of the ageing process [20]. miRNAs can modulate key ageing-related signalling pathways, such as those involving sirtuins, mTOR, and IGF-1, and are increasingly recognized as mediators of age-associated cellular homeostasis and resilience [21]. As such, a general and disease-specific dysregulation of miRNAs is seen across multiple age-related diseases, including cardiovascular disease, Alzheimer's disease, and cancer [20]. The translational value of miRNAs as regulators of ageing biology as therapeutic targets and biomarkers that can be monitored to track the ageing process is evident by an increasing body of evidence [22,23]. Their stability and ease of measurement further suggests that miRNAs could be ideal biomarkers and therapeutic targets in the context of ageing. As the field of ageing has moved into a phase of clinical translation where healthspan-prolonging interventions are garnering much hype, it is imperative to focus on mechanisms that are not only causally associated with disease but are actionable in a manner that their measurement in a clinical setting informs management decisions. Hence, we review the role of miRNAs in the biology of ageing and age-related diseases herein.

## 2 miRNAs as regulators of biological ageing

The fact that miRNA profiles change with chronological age indicates a potential mechanistic link between miRNA-mediated gene expression regulation and the ageing process. It also opens opportunities to investigate what age-associated biological processes miRNAs regulate. For example, conducting a genome-wide assessment of miRNA dynamics in peripheral blood mononuclear cells (PBMCs) with age, miR-24/-103/-107/-128/-130a/-155/-221/-496/and -1538 were significantly lower in older individuals [24]. Pathways regulated by these miRNAs included the PI3/AKT signalling pathway, c-KIT, and H2AX, suggesting a role for these processes in ageing [24]. Indeed, later studies have shown that activation of the PI3/AKT signalling pathway is a central hub of ageing biology, particularly because of its activation by IGF-1 and its downstream activation of mTOR, which are involved in ageing and loss of resilience [25]. H2AX is related to the process of DNA damage, which is central to all theories of how the ageing process is initiated [1]. Hooten et al. compared serum miRNA profiles between a cohort of young (mean age = 30 years) and old (mean age = 64 years) individuals and found that miR-151a, miR-181a and miR-1248 were significantly downregulated in old individuals [26]. Functional analyses indicated that the age-related decline in miR-1248 and miR-181a was associated with the upregulation of pro-inflammatory cytokines IL-6 and TNFα, thereby driving "inflamm-ageing" [26]. To identify miRNA signatures associated with longevity, ElSharawy et al. conducted genome-wide miRNA profiling of long-lived individuals (15 centenarians and nonagenarians; mean age 96.4 years) and younger controls (mean age 45.9 years) [27]. The analysis identified 80 differentially expressed miRNAs, with 64 being downregulated and 16 upregulated, consistent with studies demonstrating a general decrease in miRNA expression with age. After various validation procedures, miR-106a and miR-126 were confirmed to be downregulated in long-lived individuals [27]. Functionally, downregulated miRNAs were enriched in p53 and cancer-related pathways, suggesting that the upregulation of p53 with age may be a protective mechanism against tumorigenesis and perhaps a surrogate for an elevated senescent cell burden. Collectively, these studies point to an intricate link between miRNAs and key biological ageing pathways.

Recent studies, such as those by Tony Wyss-Coray's group, have found that organs age at very different rates, as indicated by their proteome and transcriptome [28,29]. Wagner et al. recently developed ncRNA expression atlas across 16 mouse tissues through the mouse lifespan and demonstrated organ-specific miRNA ageing signatures [30]. Eight miRNAs were globally differentially expressed across all tissues, suggesting their utility as potential biomarkers, especially considering that their plasma concentration mirrored their dynamics in tissues. miR-29c-3p showed the strongest correlation with ageing in solid organs, plasma, and extracellular vesicles (EVs), was functionally related to the extracellular matrix and secretion pathways, and was restored to levels comparable to those in youthful mice after heterochronic parabiosis [30]. These findings indicate that miR-29c-3p could be an ideal biomarker easily measured in serum and reflect ageing trajectories across multiple organ systems.

It is important to note that the miRNAs that change with age are common to both males and females, whereas those particular to one sex do not exhibit age-related changes (Table 1). While numerous studies report global shifts in miRNA expression with ageing, emerging evidence highlights sex-specific patterns. In over 4300 individuals, Fehlmann et al. identified 1568 miRNAs significantly correlated with age and 362 with sex, but only 231 overlapped [18]. This suggests that the dynamics of most age-associated miRNAs are not shared across sexes and that those miRNAs exhibiting sex-specific regulation tend to act independently of ageing. However, some miRNAs, such as miR-144-3p and miR-423-5p, demonstrate significant age-related expression changes in both males and females. In contrast, miR-486-5p and miR-21-5p show differential trajectories with age between the sexes, being more prominently expressed in ageing males and females, respectively. ElSharawy et al. further supported these findings by reporting that sex influenced differential regulation of several miRNAs in long-lived individuals, including miR-126, which showed stronger downregulation in aged males [17]. Moreover, downregulated miRNAs in the studies above were frequently associated with illnesses, whereas up-regulated miRNAs were less commonly described in this context [20,27]. The miRNAs that exhibited variations with ageing showed a greater degree of change at the 5′ end compared to the 3′ end [20].

**Table 1:** Sex-specific microRNA (miRNA) expression changes associated with aging.

miRNA	Expression levels with ageing	Sex-specific pattern	Reference
miR-144-3p	Increased	Increases with age in both sexes	(20)
miR-423-5p	Increased	Shared age-related increase in males and females	(20)
miR-486-5p	Decreased	Greater decline in males	(20)
miR-21-5p	Increased	More prominent increase in females	(20)
miR-126	Decreased	Stronger downregulation in aged males	(27)
miR-320b	Increased	Enriched in long-lived females	(27)
miR-222	Decreased	Greater decrease observed in males	(27)

### 2.1 DNA damage

Ageing has been conceptualized as a consequence of damage accumulation, whereby the build-up of genomic, molecular, and cellular damage is manifested phenotypically as functional decline and chronic disease. DNA damage is central to this theory, as it accumulates with age and the capacity for DNA repair declines [1]. Lopez-Otin and colleagues include DNA damage as a primary hallmark of ageing, meaning it is involved in initiating other hallmarks, most notably cellular senescence [11].

Cancer, due to the accumulation of genomically unstable cells, is the most obvious age-associated outcome associated with genomic instability, but neurodegenerative diseases and osteoarthritis have also been associated [31]. Genomic instability may be augmented in so-called premature ageing-like states, as can be seen in cancer survivors exposed to chemotherapy/radiotherapy, which can damage not only the genome of non-cancer cells but also of the surrounding, peri-tumoural tissue and also systemically [32]. This has been associated with the early onset of age-related diseases such as secondary neoplasms, neurodegeneration, and frailty compared to age- and sex-matched controls [32]. Simone et al. identified a miRNA signature in response to genotoxic agents (17 miRNAs post-irradiation, 23 post-H_2_O_2_, and 45 post-etoposide), indicating a broad miRNA response to oxidative stress and DNA damage [33].

**Fig. 2:**
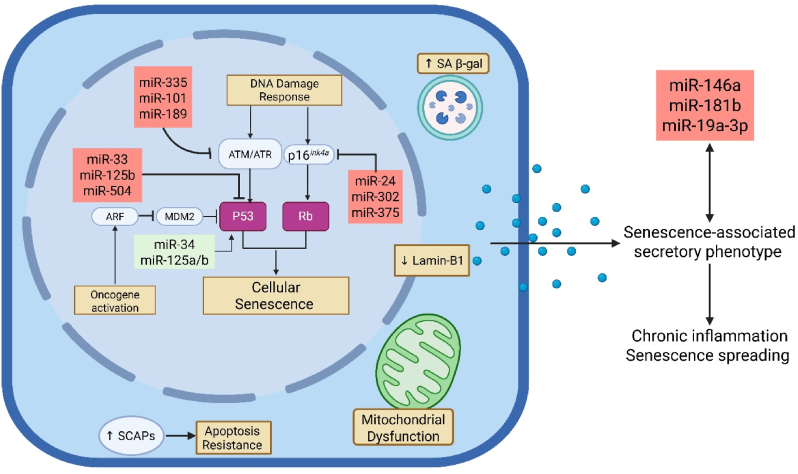
**The role of miRNAs in regulating the DNA damage response and cellular senescence.** The DNA damage response can be triggered by genotoxic stress or telomere attrition but can also result from many cellular stressors. Regardless of the stimulus, the DNA damage response converges on the activation of either Rb or p53, which induces cell cycle arrest. This may lead to DNA repair followed by a resumption of replication, but it could lead to cellular senescence, a permanently arrested state. Cellular senescence has the following cellular hallmarks: elevated SA β-gal activity, reduced lamin-B1, mitochondrial dysfunction with high ROS production, and upregulation of senescence-associated anti-apoptotic pathways (SCAPs), which render senescent cells apoptosis-resistant. As shown in the figure, miRNAs interact with multiple steps in this process.

The DNA damage response is a multi-step process illustrated in Fig. 2. It starts with the activation of kinases like ATM and ATR, the phosphorylation of H2AX (forming γH2AX) to mark DNA damage foci and remodel surrounding chromatin to allow enzymes and repair proteins access to damaged regions, cell cycle arrest by proteins such as p16^*ink4a*^/Rb and p21/p53, and then several forms of repair depending on the type of DNA damage [34]. Double-stranded breaks are repaired by homologous recombination or non-homologous end-joining, whereas single-stranded DNA breaks are repaired by base excision repair, nucleotide excision repair, or mismatch repair. The DNA damage response is triggered by the activation of protein kinases such as ATM and ATR, which phosphorylate and activate CHK1/2. These then activate p53 by phosphorylation, which triggers cell cycle arrest by inhibiting CDK2. P53 also activates MDM2, inhibiting p53 and setting up a negative feedback loop. The activation of oncogenes induces p53 via an alternate mechanism through activation of ARF2, which inhibits MDM2 as a method of activating p53. The outcome of p53 activation is either successful DNA repair followed by a resumption of the cell cycle, unsuccessful repair followed by apoptotic cell death, or cellular senescence, a form of cell cycle arrest that is accompanied by profound changes in cell morphology, gene expression, and secretory phenotype. DNA damage also activates the expression of the *CDNK2A* gene encoding p16^*ink4a*^, which inhibits CDK4/6. This leads to hypo-phosphorylation of retinoblastoma (Rb) protein, which mediates cell cycle arrest. Other reviews provide in-depth explanations of the DNA damage response [34].

MiRNAs regulate several important proteins involved in this process. Table 2 summarises studies on the role of miRNAs in the DNA damage response and cellular senescence. Fig. 2 depicts miRNAs known to regulate key steps in the DNA damage response. For example, miR-18a, miR-26b, the miR-30 family, miR-100, miR-203, miR-223, and miR-421 all inhibit the translation of ATM by binding to the 3′-UTR of its mRNA, impairing the repair of DNA double-stranded break [[35], [36], [37], [38]]. This mechanism may be involved in escaping cell cycle arrest seen in multiple tumours. Similarly, miR-138 and miR-24 target histone H2AX, suppressing its expression and increasing DNA damage response in response to cytotoxic anticancer therapies [39].

**Table 2:** Mechanistic links between miRNAs and DNA damage response/cellular senescence.

miRNA	Association with DNA Damage or Senescence	Reference
miR-335	Downregulated by ATM activation following irradiation-induced DNA double-strand breaks (DSBs), affecting CtIP levels and homologous recombination repair (HRR).	(172)
150 miRNAs	Show altered expression following DNA damage, with changes regulated by p53 in a cell-type-specific manner; some p53-regulated miRNAs correlate with cancer patient survival.	(41)
miR-106b family	Overrides doxorubicin-induced DNA damage checkpoint, promoting cell cycle progression by targeting p21/CDKN1A.	(40)
miR-34a	Induced by p53 activation following DNA damage, leading to apoptosis and G1-arrest, suppressing tumour proliferation.	(173)
miR-34	Loss-of-function mutations cause abnormal cellular survival responses to radiation in *C. elegans* (radiosensitivity in soma, radioresistance in germline); exogenous addition alters survival post-radiation in breast cancer cells.	(174)
miR-101	Targets ATM, sensitizing tumour cells to radiation by impairing DNA repair.	(175)
miR-16	Negatively regulates Wip1, delaying its increase to ensure proper DNA damage response.	(176)
miR-18a	Upregulated in breast cancer, it downregulates ATM by targeting its 3′-UTR, impairing DNA damage repair and increasing radiosensitivity.	(177)
miR-34	Inactive pool lacking 5′-phosphate is rapidly activated by DNA damage through 5′-end phosphorylation (ATM- and Clp1-dependent), enabling loading into Ago2.	(178).
miR-15b/16-2	miR-15b is upregulated post-irradiation, and induces p53 phosphorylation, DNA repair, apoptosis, and cell cycle arrest while inhibiting Wip1 expression.	(179)
miR-34 family	Directly activated by p53, induced by DNA damage and oncogenic stress, suppresses cell proliferation by targeting cell cycle progression genes.	(180)
miR-21	Downregulates hMSH2 and hMSH6, reducing 5-FU-induced G2/M arrest and apoptosis, leading to resistance in colorectal cancer cells.	(181)
miR-504	Negatively regulates p53 by binding its 3′-UTR, reducing p53-mediated DNA damage responses (transcriptional activity, apoptosis, cell-cycle arrest).	(43)
miR-125b	Upregulated by UV radiation via ATM-dependent NF-κB activation, represses p38α, promoting cell survival and protecting against UV-induced apoptosis.	(182)
miR-339-5p	Targets MDM2, reducing its expression and promoting p53 function in response to stress, enhancing proliferation arrest and senescence.	(44)
miR-1255b, miR-148b∗, miR-193b∗	Suppress homologous recombination in G1 phase by targeting BRCA1, BRCA2, and RAD51, impairing DNA repair and increasing genomic instability.	(183)
miR-107	Activated by p53 via PANK1, downregulates CDK6 and p130, affecting G1/S progression in response to doxorubicin-induced DNA damage.	(184)
miR-605	Activated by p53 in response to stress, represses Mdm2, forming a positive feedback loop to enhance p53 activity and induce apoptosis.	(185)
let-7a-1, miR-16-1, miR-145, miR-34a	BRCA1 accelerates their processing in response to DNA damage, increasing precursor and mature forms, linked to tumour suppression.	(186)
miR-34a	Represses HDM4, creating a positive feedback loop with p53 to enhance its activity in response to DNA damage, contributing to tumour suppression.	(187)
miR-210, miR-373	Upregulated in hypoxic conditions, suppress RAD52 and RAD23B, impairing DNA repair pathways (HDR and NER) and contributing to genetic instability.	(188)
miR-29, miR-30	Up-regulated during senescence in an Rb-dependent manner, repress B-Myb expression, promoting Rb-driven senescence	(189)
miR-26b, miR-181a, miR-210, miR-424	Promote senescence by repressing Polycomb Group proteins and activating p16^*ink4a*^	(190)
miR-15, miR-106b family, miR-182, miR-183	miR-15 and miR-106b are downregulated, while miR-182 and miR-183 upregulated in stress-induced premature senescence in human diploid fibroblasts and human trabecular meshwork cells *in vitro*	(191)
miR-22	Up-regulated in senescent cells, targets CDK6, SIRT1, and Sp1 to induce senescence and suppress proliferation	(192)
miR-34a	Increases with senescence, induces endothelial senescence by targeting and suppressing SIRT1	(193)
miR-221/222	Up-regulated in senescent fibroblasts, associated with G1/S cell cycle arrest	(194)
miR-499, miR-34c	Potential to regulate all four senescence induction types (telomere attrition, oxidative stress, oncogene expression, DNA damage)	(195)
miRNAs (general)	Regulate cellular senescence and inhibit insulin/IGF1 and TOR signaling pathways, modulating lifespan	(196)
miR-210, miR-376a∗, miR-486-5p, miR-494, miR-542-5p	Upregulated during replicative and chemically induced senescence, induce senescence markers, DNA damage, and ROS accumulation	(197)
Let-7g, miR-26a miR-136 miR-144 miR-195 miR-200b, others	All upregulated in senescent states but not in quiescent state. miR-34a. Let-7f and miR-34a are strongly upregulated in stress-induced premature senescence (SIPS), and miR-638 and miR-663 in replicative senescence	(198)
miR-34a	Up-regulated in response to DNA damage, induces senescence-like phenotypes by modulating the E2F pathway	(199)
miR-760, miR-186, miR-337-3p, miR-216b	Up-regulated in replicative senescence, cooperatively induce senescence via CK2Î± downregulation and ROS-p53-p21 pathway	(200)
miR-33	Promotes replicative senescence in MEFs by suppressing CDK6	(201)
miR-17-92 cluster	Decreased expression linked to senescence features and reduced MSC therapeutic efficacy; modification reverses senescence	(202)
miR-483-3p	Up-regulated during *in vitro* passaging, induces senescence in hADSCs by targeting IGF1	(203)
miR-59	Up-regulated in HGPS cells, inhibition alleviates cellular senescence	(204)
miR-17, miR-20a	Decreased in senescent MSCs, linked to c-Myc stemness and p21 expression; modification reverses senescence	(205)
miR-101	Induces mild/moderate DNA damage leading to senescence rather than apoptosis by targeting UBE2N and SMARCA4	(206)
miR-155-5p	Down-regulated in senescent hVSMCs and exosomes, linked to TGF-Î^2^/NF-Î°B signaling and cell cycle arrest	(207)
miR-494-3p	Up-regulated in oxidative stress-induced senescence, increases senescence markers and SASP proteins by targeting SIRT3; inhibition reduces senescence	(208)
let-7b, miR-101	Induced in senescence, target EZH2; KDM2B silences them to bypass senescence	(209)
miR-25, miR-26a, miR-27b, miR-92a, miR-7, let-7	E2F7 negatively regulates these proliferation-promoting miRNAs; let-7 maturation modulated by E2F/c-MYC/LIN28B axis	(210)
miR-34a	Induces senescence in endothelial cells by targeting Bcl-2	(211)
miR-217	Up-regulated in endothelial cells, promotes senescence by targeting SIRT1	(212)
miR-200c	Up-regulated in senescent endothelial cells, targets ZEB1, promoting senescence	(213)
miR-125b	Promotes senescence in malignant melanoma to restrain cell proliferation	(214)
miR-21	Up-regulated in senescent cells, promotes senescence by targeting PTEN	(215)
miR-24	Down-regulated in senescent fibroblasts, inhibits senescence by targeting p16	(216)
miR-203	Up-regulated in senescent keratinocytes, induces senescence by targeting p63	(217)
miR-449a	Up-regulated in senescent cells, targets cyclin D1, promoting senescence	(218)
miR-216a	Inhibits Smad3 to induce senescence in endothelial cells	(219)
miR-199a	Inhibits endothelial cell senescence under diabetic (high glucose) conditions by targeting DDR1 to inhibit DDR1/p21/p53 signalling	(220)
miR-335	Upregulated in cancer cells and activates the p53 pathway to promote senescence	(221)
miR-192, miR-215	Upregulated by p53 and enhance p21 levels to promote senescence and suppress carcinogenesis	(222)
miR-29b	Upregulated by TGF-β, miR-29a/c promote senescence by suppressing Suv4-20h, reducing H4K20me3, and impairing DNA damage repair	(223)
miR-106a	Downregulated in senescent cells, it inhibits senescence by targeting p21	(191)
miR-20a	Induces senescence by inhibiting the proto-oncogene *LRF,* increasing p19ARF and p16^*ink4a*^ while decreasing E2F1	(224)
miR-92a	Downregulated in senescent cells, linked to proliferation inhibition and senescence	(225)
miR-181b	Upregulated in response to oxidative stress and induces senescence in endothelial cells	(226)
miR-34b	Inhibits *MYC* independently of p53 to promote oncogene-induced senescence	(227)
miR-146b	Upregulated in senescent cells, modulates SASP by targeting IRAK1	(69)
miR-155	Upregulated in aged MSCs and promotes senescence by inhibiting mitochondrial fission and Cab39/AMPK signalling, while its downregulation rejuvenates MSCs	(228)
miR-195	Upregulated in ageing myoblasts and promotes senescence by inhibiting SIRT1 and TERT	(229)
miR-302	Reverses cellular senescence by targeting *CDKN1A and CCNG2*, restoring proliferation and promoting rejuvenation in ageing mice	(230)
miR-23a	Upregulated in senescent fibroblasts, miR-23a promotes senescence by targeting hyaluronan synthase 2	(231)
miR-23a	Induces replicative senescence by targeting telomere repeat binding factor 2 (TRF2), thereby causing telomere attrition	(232)
miR-17-S3p	Attenuates cardiac senescence by targeting Par4, enhancing the self-renewal capacity of cardiac fibroblasts	(233)
miR-93	Inhibits cellular senescence by targeting Bcl-w and p21	(234)
miR-214	miR-214-3p contain in MSC-derived extracellular vesicles downregulate ATM/p53/p21 signalling to attenuate senescence and ameliorate radiation-induced lung injury	(235)
miR-494	Downregulated in senescent osteocyte-derived exosomes, leading to inhibited osteogenic differentiation and accelerated age-related bone loss. Its overexpression rescues these effects by PTEN inhibition and activation of the PI3K/AKT signalling pathway	(236)
miR-376a/b	Upregulated in Hutchinson-Gilford Progeria syndrome fibroblasts, both promote senescence	(237)
miR-30a	miR-30a promotes senescence and inhibits autophagy by targeting Beclin1, and these effects can be inhibited by rapamycin treatment of vascular smooth muscle cells	(238)
miR-34c	In acute myeloid leukemia stem cells, miR-34c promotes p53/p21^*cip1/waf1*^-CDK/cyclin pathways to induce senescence and inhibit leukemia development	(239)
miR-186, miR-216b, miR-337-3p miR-760	When upregulated in colon cancer cell lines, these miRNAs increase SA β-gal staining, p53/p21^*cip1/waf1*^ expression and ROS production, whereas their knockdown suppressed senescence	(240)
miR-1204 miR-663 let-7 family miR-519a	In human diploid fibroblasts that have undergone replicative senescence, let-7 family miRNAs are downregulated, while miR-1204, miR-663, and miR-519 are upregulated. miR-519 promotes senescence by targeting the RNA-binding protein HuR	(241)
miR-130a	Upregulated by metformin in high-glucose-exposed renal tubular epithelial cells. Metformin reduced senescent cell burden by upregulating miR-130a, which reduced STAT3 expression. This axis may reduce senescent cell burden in renal tubular epithelium in mouse models of diabetic nephropathy	(242)
miR-29c miR-369 miR-371 miR-499 let-7f	Upregulated in mesenchymal stromal cells (MSCs) upon serial replication and replicative senescence and contribute to the altered phenotype and reduced differentiation potential of these cells	(243)
miR-10a	Downregulated with ageing of MSCs. Overexpression of miR-10a increased differentiation capacity of MSCs and reduced cellular senescence by suppressing KLF4, while its knockdown has the opposite effect	(244)
miR-23b	Enriched in extracellular vesicles (EVs) derived from aged mice and promoted cellular senescence by targeting *Tnfaip3* gene expression. Exogenously administering miR-23b led to worsening of SASP-mediated liver inflammation	(245)
miR-152 miR-181a	Upregulated in human dermal fibroblast senescence and their overexpression is sufficient to induce senescence. This was associated with disrupted expression of cell adhesion and extracellular matrix proteins integrin-α and collagen XVI, which may contribute to skin ageing	(246)
miR-543 miR-590	Downregulation in ageing mesenchymal stromal cells leads to upregulation of aminoacyl-tRNAsynthetase-interacting multifunctional protein (AIMP)/p18, which induces cellular senescence in MSCs and compromises replicative and adipogenic differentiation potential.	(247)
miR-885	Induces senescence by targeting *CDK2* and mini-chromosome maintenance protein-5 (*MCM5)*, thereby activating p53 to induce cell cycle arrest followed by senescence or apoptosis	(248)
miR-370 miR-1236	Can be upregulated in various cancer cell lines, where they induce senescence by targeting cyclin D1-CDK4/6 axis and activating p21 expression	(249)
miR-138	Downregulated in renal cell carcinoma (RCC) cell lines. Its upregulation silences EZH2 expression and increases p16^*ink4a*^ expression, resulting in cell senescence	(250)
miR-27a	Significantly downregulated in coronary heart disease (CHD) patients. miR-27a overexpression in endothelial cells reduces oxidative stress, thereby reducing senescence markers SA β-gal and γH2AX	(251)

miRNAs also regulate the proteins that induce cell cycle arrest. The miR-106 family suppresses the translation of p21 [40]. Hattori et al. reported that 150 miRNAs showed altered expression following the DDR, which were regulated by p53 in a cell-type-specific manner [41]. miR-125b and miR-504 inhibit p53 expression, impairing DNA damage responses and its outcomes such as apoptosis or senescence [42,43]. Conversely, miR-339-5p targets MDM2, a negative regulator of p53, thereby promoting cell cycle arrest and cellular senescence [44].

The miR-29 family plays a significant role at the intersection of DNA damage, ageing, and cellular senescence. Levels of miR-29c-3p increase with age, and in old mice, these levels are lowered to levels comparable to youthful levels after heterochronic parabiosis—the transfer of plasma from young to old mice [30]. Expression of the miR-29 family is increased in a p53-dependent manner in Zmpste24-deficient mice, a model for Hutchinson-Gilford progeria syndrome characterized by accelerated ageing secondary to premature DNA damage accumulation. Indeed, miR-29 expression is triggered by p53 in response to DNA damage [45]. Mechanistically, miR-29c-3p suppresses phosphatase Ppm1d Wip1), a negative regulator of the p53 pathway, thereby enhancing p53 phosphorylation and activation. This creates a feedforward loop that stabilizes p53 and reinforces cellular senescence. In fibroblasts, miR-29 overexpression reduces proliferation and increases γH2AX foci and senescence-associated β-galactosidase (SA β-gal) activity, all hallmarks of cellular senescence. Conversely, inhibition of miR-29 delays senescence and restores proliferative capacity. Ding et al. reported that the age-related increase in miR-29 drives senescence of mesenchymal stromal cells (MSCs), which is associated with reduced cortical bone density and trabecular bone mass. In contrast, its genetic knockdown reduces the burden of senescent MSCs, enhances bone mass, and accelerates calvarial defect regeneration [46].

### 2.2 Telomere attrition

In 1961, Hayflick and Moorehead observed that fibroblasts did not divide infinitely *in vitro* but stopped dividing after a set number of replicative cycles. This was termed the ‘Hayflick Limit’ and was later understood to be due to telomere shortening (or attrition) [47]. Telomeres are redundant segments of non-coding DNA present at the ends of chromosomes, and they shorten with successive cell division. When cells reach their ‘Hayflick Limit’, the lack of adequate telomere length leads to the loss of DNA with cell division, triggering a DNA damage response and cellular senescence (termed ‘replicative’ senescence). It has long been known that telomeres shorten with age, and this has been associated with a variety of age-related outcomes such as cancer, osteoporosis, osteoarthritis, and all-cause mortality [48]. However, the promise of elongating telomeres to improve healthspan has not yet been realized. Telomerase is the enzyme that can elongate telomeres but carries a recognized risk of promoting oncogenesis, as telomerase expression constitutes one of the principal mechanisms through which cancer cells acquire replicative immortality.

In keeping with the context of the review, studies have explored the role of miRNAs as mediators of telomere dysfunction. POT1 is a protein that may play a role in protection against DNA damage. MiR-185 binds to the POT1 3′-UTR, decreasing its translation into protein and thereby precipitating telomere dysfunction [49]. Additionally, telomeric repeat binding factor 2 (TRF2) is important for the preservation of telomeres. Inhibition of TRF2 by miRNA-23a can cause telomere disruption and thereby accelerate cellular senescence in human fibroblasts, which could be rescued by ectopically expressing TRF2 [50]. Lastly, a decrease in miR-122 expression levels has been shown to inhibit NF-κB-dependent inflammatory responses by attenuating cellular senescence [51].

### 2.3 Cellular senescence

Cellular senescence describes a state of permanent cell cycle arrest that cells undergo in response to various intrinsic and extrinsic stressors, such as DNA damage, strong mitogenic stimuli including the activation of oncogene or silencing of tumour-suppressor genes, mitochondrial dysfunction, mechanical/shear stress, and extracellular signals [52]. Senescent cells adopt a strong secretory phenotype, secreting a senescence-associated secretory phenotype (SASP) that reinforces the senescent state via autocrine effects and induces senescence in neighbouring cells through paracrine and endocrine effects [32,53]. The SASP also activates immune cells and releases proteases that remodel the local microenvironment, leading to chronic inflammation and tissue/organ dysfunction. Typically, senescent cells are eliminated by immune cells. However, when the number of senescent cells surpasses a certain threshold, likely due to declining immune function with age, these cells accumulate, damage tissues, and contribute to the onset of various chronic diseases [54,55].

The p16ink4a/Rb axis, p53/p21^*cip1/waf1*^ axis, and other mechanisms regulate cellular senescence (Fig. 2). These mechanisms and SASP factors (e.g., cytokines, growth factors, lipoxins, and proteases) can identify senescent cells *in vivo*. Recently, a minimal criteria for identifying senescent cells was established to standardize their definition and enable better comparisons across different contexts [56]. miRNAs are essential components of the SASP that can amplify inflammatory reactions and induce senescence in neighbouring non-senescent cells [57]. A preprint study profiled the landscape of miRNA across different human cell types. It demonstrated that they could effectively track senescent cell burden across ageing and after interventions that eliminate senescent cells. A list of 22 such “senomiRs” was compiled to serve as biomarkers of senescence to be investigated in ongoing senotherapeutic clinical trials [58].

The interaction between miRNAs and the cellular senescence regulators, including p16^*ink4a*^, p21^*cip1/waf1*^, and p53, is complex and can either lead to senescence induction or inhibition. Hundreds of studies link different microRNAs to suppressing or inducing cellular senescence. We have attempted to summarize many of these in Table 2. To list a few examples, miR-24 inhibits replicative senescence by suppressing p16^*ink4a*^ expression in human skin fibroblasts [59]. miR-217 suppresses DNA methyltransferase 1 (DMNT1) expression, upregulating p16^*ink4a*^ mRNA and inducing senescence in human skin fibroblasts [60]. In human mammary epithelial cells, transcriptional activation of p16^*ink4a*^ by miRNAs such as miR-26b and miR-181a induces senescence by targeting the Polycomb group proteins, a group of repressive protein complexes that act via chromatin modification [61]. Conversely, miR-25, miR-30d, and miR-125b prevent senescence by directly binding to p53 mRNA and inhibiting its expression at the transcriptional level [62]. Under oxidative stress, miR-34a reduces the expression of gene *Sirt1*, thereby maintaining the stability of p53, leading to the induction of cellular senescence [63]. miR-20b-5p significantly inhibits the expression of p21^*cip1/waf1*^, thereby promoting cell proliferation in human multipotent stromal cells under oxidative stress [64]. miR-124, miR-34a, and miR-29a/b/c can block cyclin A2, which represents a distinct senescence-inducing mechanism [65].

Besides mediators of the DNA damage response, miRNAs regulate cellular senescence in response to various extrinsic signals. For example, the TGF-β/Smad signalling pathway is key in controlling stress-induced and developmentally planned senescence [66]. H4K20me3, a target of classical TGF-β signalling, is downregulated by miR-29. Lyu et al. reported that canonical TGF-β signalling secondary to oxidative stress upregulates miR-29a/c, which silences expression of Suv4-20h, thereby reducing H4K20me3 levels and impairing DNA repair. The resulting H4K20me3 loss, driven by the TGF-β/miR-29 axis, contributes to cardiac aging *in vivo* while blocking TGF-β signalling restores H4K20me3 levels and enhances cardiac function in aged mice.

MiRNAs also regulate the SASP. Upregulation of miR-216a in human umbilical vein endothelial cells suppresses the expression of small mothers against decapentaplegic 3 (Smad3), which affects the breakdown of NF-KB inhibitor alpha and the activation of adhesion molecules [67]. Senescent dermal human fibroblasts secrete extracellular vesicles as part of the SASP, which contain several miRNAs, most of which target mRNAs of pro-apoptotic proteins. This suggests that miRNAs in the SASP could contribute to the maintenance of senescence features via cell-autonomous/non-cell-autonomous effects [68]. Judith Campisi and colleagues reported that miR-146a/b were significantly upregulated in senescence as a compensatory response to the SASP and restrain inflammation by suppressing IL-6 and IL-8 secretion and downregulating *IRAK1*, a component of the IL-1R signal transduction pathway [69]. These findings indicate that miR-146a/b is expressed in response to the pro-inflammatory SASP and functions as part of a negative feedback loop to restrain excessive inflammation. These findings were replicated *in vivo* in mouse models of tendinopathy, where miR-146a expression reduced SASP-mediated inflammation and ameliorated disease progression [70]. Other studies have shown that miR-146a increases with ageing and features in cellular senescence and SASP-mediated inflammation across multiple tissue types [[71], [72], [73]]. For instance, dysregulated expression of miR-146a-5p may amplify inflammation and senescence-related processes in healthy cells. RNA sequencing analysis of visceral adipose tissue from df/df mice revealed that elevated levels of miR-146a-5p suppress apoptotic signalling pathways [57].

From a clinical perspective, the value of cellular senescence lies in the prospect of selectively eliminating senescent cells by senolytic agents or attenuating their pro-inflammatory phenotype by senomorphic drugs. These classes of drugs are called senotherapeutics or senotherapies. The first-generation senolytic agents include dasatinib, fiscetin and quercetin, which are being studied in multiple clinical trials [74]. Leveraging the observation that miR-181b declines with age and targets many factors associated with cellular senescence and the SASP, Moreno et al. transfected old and young human aortic smooth muscle cells with miR-181b [75]. Upon transfection, miR-181b reduced expression of p16^*ink4a*^ and p21^*cip1/waf1*^ and markers of the SASP, which was associated with greater proliferation of smooth muscle cells and lower arterial vessel stiffness as compared to old, untreated cells. Kaur et al. reported that miR-19a-3p decreases with age in bone samples of mice and humans [76]. miR-19a-3p expression further decreased with senescence induction in bone marrow stromal cells, while its overexpression in osteoblasts suppressed senescence markers p16^*ink4a*^ and p21^*cip1/waf1*^. Importantly, inducing senescence in miR-19a-3p-expressing cells attenuated expression of senescence markers (p16^*ink4a*^, p21^*cip1/waf1*^, and SA β-gal), suggesting that its expression can effectively alleviate senescence phenotypes [76]. Combining senolytic agents and miRNA-based therapeutics as an approach to enhance elimination of senescent cells was demonstrated by Herman et al., who showed that overexpression of miR-340-5p can reinforce a senescent phenotype and thereby enhance sensitivity to senolytic compounds [77].

### 2.4 Mitochondrial dysfunction

The mitochondria are responsible for more than just cellular ATP production. They regulate calcium homeostasis, the redox state of the cell, and apoptosis. Mitochondrial dysfunction includes reduced mitochondrial oxidative capacity, lower mitochondrial membrane potential, and/or elevated mitochondrial reactive oxygen species (ROS) production [78]. The mitochondria also represent a key regulator of inflammation, and their dysfunction activates pathways such as the NLRP3 inflammasome and cGAS/STING [79,80]. These pathways regulate cellular senescence induction and the SASP [81]. Mitochondrial dysfunction with elevated ROS production is also one of the consequences of cellular senescence [82]. Conversely, mitochondrial dysfunction can induce cellular senescence with a distinct SASP lacking IL-1-dependent inflammation [83]. In the context of miRNAs, a decrease in miR-15b leads to the induction of SIRT4, thereby increasing mitochondrial ROS production and inducing senescence and SASP expression [84].

miRNAs regulate several aspects of mitochondrial dynamics (fission, fusion, mitophagy, ATP production, oxidative stress, and apoptosis) critical for maintaining organ function [85,86]. Multiple nuclear-encoded pre-miRNA and mature miRNAs, including miR-1 and miR-181c, along with other miRNA-related machinery such as argonaute-2 (AGO2), translocate from the cytosol to the mitochondria and directly regulate the translation of mitochondrial genes, [[87], [88], [89], [90]].

miRNAs regulate cardiac remodelling and hypertrophy, and at least part of this effect may be due to their modulation of mitochondrial function. Administering miR-181c leads to reduced exercise capacity and signs of heart failure in animal models, indicated by reduced left ventricular fractional shortening and ejection fraction, associated with inhibition of *COX1* [91]. In the heart, this was related to elevated levels of miR-181c in the mitochondria, reduced expression of complex IV of the electron transport chain and increased oxygen consumption [91]. miR-214 can suppress SIRT3, leading to mitochondrial dysfunction, cardiac hypertrophy and eventual failure. Overexpression of miR-214 exacerbated mitochondrial damage and facilitated cardiac hypertrophy, whereas genetic knockdown of miR-214 restored SIRT3 expression and attenuated mitochondrial dysfunction [92].

Several miRNAs are differentially associated with sarcopenia [93]. miR-181a targets autophagy-related genes such as *PRKN* and *PAR7* (encoding mitophagy-promoting proteins parkin and DJ-1, respectively) and mediates mitochondrial fission [94]. Its downregulation disrupts mitochondrial fission, leading to fragmented mitochondria, reduced muscle mitochondrial content, and ageing phenotypes such as loss of muscle mass. Conversely, restoring miR-181 expression enhances mitophagy, clears damaged mitochondria, and improves muscle strength [94]. In denervated muscles, the process of atrophy is partly mediated by mitophagy activation via the miR-142a-5p/mitofusin axis [95].

In AD, miR-455-3p transgenic mice exhibit increased mitochondrial biogenesis (elevated PGC1α, NRF1/2, TFAM), improved synaptic activity (higher SNAP25, PSD95, MAP2), and extended lifespan, while knockout mice display reduced mitochondrial numbers and cognitive deficits [96]. In contrast, miR-140 is upregulated and suppresses *PINK1* to increase ROS and reduce mitochondrial membrane potential. At the same time, its silencing enhances autophagy via the mTOR pathway and improves cognitive function, while its overexpression exacerbates neural damage and mitochondrial dysfunction [97]. In Parkinson's disease brain samples, miR-34b/c expression is decreased early in the disease course in areas like the amygdala, frontal cortex, substantia nigra, and cerebellum [98]. In dopaminergic neuronal cell lines, this downregulation is associated with mitochondrial dysfunction, as indicated by downregulation of *PRKN* and *PARK7* (both of which are known to regulate mitophagy and are mutated in autosomal recessive forms of Parkinson's disease)*,* oxidative stress, reduced cell ATP content, and decreased cell viability [98]. These findings indicate that early downregulation of miR-34b/c in Parkinson's disease may lead to downstream transcriptome alterations that may render mitochondria dysfunctional and compromise cell viability.

miRNAs also regulate mitochondrial stress responses, such as apoptosis and ROS production. Li et al. report that miR-145 is downregulated oxidative stress-induced apoptosis in cardiomyocytes in ischemic/reperfusion injury *in vivo* or H_2_O_2_ treatment *in vitro* [99]. Overexpressing miRN-145 attenuated cardiomyocyte apoptosis, whereas its inhibition increased apoptosis. Mechanistically, miR-145 inhibits expression of Bnip3, thereby reducing ROS production, preserving the mitochondrial structure and decreasing expression of mitochondrial apoptotic markers such as cytochrome *c* release and Apaf-1, highlighting that preservation of mitochondrial structure and function by miRNA can increase the resilience of cardiomyocytes to stressors [99]. On the other hand, miR-181a augments H_2_O_2_-induced cardiomyocyte apoptosis by modulating the mitochondrial apoptotic pathway [100].

Bai and colleagues investigated the dynamics of miRNAs in ageing rat kidneys [101]. Compared to young rat kidneys, 18 miRNAs were upregulated, and seven miRNAs were downregulated in 24-month-old rat kidneys. Of these differentially expressed miRNAs, miR-34a and miR-335 were significantly upregulated in renal mesangial cells. Both of these miRNAs inhibit the expression of the antioxidative enzymes superoxide dismutase-2 (SOD2) and thioredoxin reductase 2 (Txnrd2) located in the mitochondria, consequently increasing ROS production and inducing cellular senescence. In agreement with these findings, overexpression of miR-34a and miR-335 induced premature senescence in mesangial cells, whereas their inhibition by their respective antisense miRs inhibited cellular senescence and attenuated mitochondrial dysfunction [101].

MiRNAs may regulate mitochondrial dysfunction as a component of the biology of metabolic syndrome. For example, an intron region of the peroxisome proliferator-activated receptor γ coactivator 1β (PGC-1β), a transcriptional coactivator, encodes miR-378/378∗. These miRNAs inhibit key elements of mitochondrial function, such as carnitine-O-acyltransferase, an essential mediator of fatty acid metabolism [102]. miR-378/378∗-deficient mice resist high-fat diet-induced obesity, and exhibit enhanced mitochondrial oxidative and fatty acid metabolism capacity in insulin-sensitive tissues [102]. In another study, mice overexpressing hepatic miR-378/378∗ share phenotypic similarities to mice deficient in insulin signalling, while their knockout enhances insulin sensitivity [103]. miR-149 is downregulated in HFD-fed obese mice, leading to increased PARP-2, reduced NAD^+^, and impaired mitochondrial biogenesis, while its overexpression boosts NAD^+^ levels, SIRT-1 activity, and mitochondrial content [104]. Conversely, HFD-induced miR-149 suppression exacerbates mitochondrial dysfunction, underscoring miR-149's role in maintaining skeletal muscle energy homeostasis [104]. miR-141-3p is upregulated in HFD-induced obese mouse livers, increasing ATP production and oxidative stress by targeting PTEN [105]. Inhibiting miR-141-3p or PTEN silencing reverses these effects, highlighting miR-141-3p′s role in disrupting mitochondrial function in obesity-related metabolic disorders via PTEN suppression.

Collectively, these findings show that the modulation of mitochondrial structure and function constitutes a principal way miRNAs play a role in the pathogenesis of various age-related diseases.

## 3 Role of miRNAs in age-related morbidities

### 3.1 Geriatric syndromes

Patients diagnosed with sarcopenia exhibit significant muscle mass loss, which is associated with alterations in circulating miRNAs. MiR-486 exhibits a high level of expression in skeletal muscle and directly affects Pax7, hence facilitating the differentiation of myoblasts. A decrease in miR-486 levels is seen in sarcopenic older individuals, which may be a factor in the progressive loss of muscle mass. Additionally, levels of miR-146a are decreased in sarcopenic individuals compared to healthy controls [106].

The fact that older individuals cannot build muscle mass as well as their younger counterparts after resistance exercise may also contribute to age-related sarcopenia. Exploring whether miRNAs played a role in this reduction of plasticity, Rivas et al. demonstrated that 21 miRNAs were differentially expressed following resistance exercise in young men. At the same time, none were altered in older men. This was associated with altered transcription of 175 genes in young men, compared to only 42 genes in old men [107]. Central to this response to exercise was miR-126, which was downregulated in younger men post-exercise but unaltered in older men, contributing to blunted mRNA transcription and reduced lean mass. Manipulating miR-126 expression in myoblasts alters IRS1, FOXO1, and other myogenic factors involved in skeletal muscle growth, highlighting its role in the anabolic response in ageing muscle. Conversely, the absence of miR-126 regulation in older men exacerbates sarcopenia by limiting muscle growth pathways.

Frailty is a geriatric syndrome characterized by a decreased physiological reserve and a greater vulnerability to stressors, leading to a reduced ability to maintain homeostasis. Two studies have reported several miRNAs that could potentially serve as new candidate biomarkers for frailty. A study examined alterations in the plasma-derived exosome miRNA profiles of young, old robust, and old frail persons, identifying eight miRNAs specifically dysregulated in the frail group [108]. Studying the profiles of three miRNAs associated with inflammation and one miRNA involved in regulating melatonin production in the blood of healthy people, older individuals with good health, and older individuals with fragile health, miR-21 had a greater level of expression in frail individuals compared to controls [109]. A comprehensive analysis of RNA extracted from whole blood detected nine miRNAs differentially expressed in frail individuals compared to healthy controls. For instance, two specific miRNAs, miR-101-3p and miR-142-5p, were consistently downregulated in frail participants compared to healthy subjects [110].

### 3.2 Cardiometabolic disorders

Alterations in miRNA levels have demonstrated high sensitivity for congestive heart failure (HF) [111]. Specific miRNAs are highly expressed CD15^+^ granulocytes, and these cells play a role in the inflammatory response throughout the development and progression of HF [20,112]. Sampling plasma from individuals with HF reveals dysregulation of six miRNAs, some of which are referenced in Table 3 [112]. Similarly, cardiac specimens from individuals with non-ischemic HF and systolic HF demonstrate dysregulated miRNAs in cardiomyocytes [111]. The levels of these miRNAs in the plasma positively correlated with troponin T levels, suggesting that miRNAs were released from damaged cardiomyocytes [113,114]. Additionally, miRNAs may contribute to altering the heart's structure and electrical characteristics, suggesting their role in the development of some arrhythmias. For example, the expression of miRNA-150 is significantly decreased in patients diagnosed with atrial fibrillation (AF) [115].

**Table 3:** Association of select miRNAs with age-related disorders.

miRNA	Expression levels	Target tissue	Major finding	Biological Function	Reference
miR-622	Increased	Heart (myocardium)	Elevation in the myocardium in case of non-ischemic and systolic HF	miR-622 likely contributes to the progression of systolic heart failure by targeting genes involved in cardiac muscle contraction, MAPK, and Notch signalling, thereby influencing myocardial remodelling and contractility	(111)
miR-423-5p	Increased	Heart	Significantly related to the clinical diagnosis of HF	miR-423-5p is functionally linked to heart failure through its role in cardiac remodelling and stress responses, and has been proposed to influence myocardial hypertrophy and fibrosis by modulating cardiomyocyte signalling pathways	(111)
miR-21, miR-29b, and miR-1	Increased	Heart	Highly elevated in acute myocardial infarction (MI)	miR-1, miR-21, and miR-29b contribute to myocardial infarction pathology by regulating cardiomyocyte apoptosis and electrophysiology (miR-1), promoting cardiac fibroblast activation and fibrosis (miR-21), and modulating extracellular matrix remodelling through suppression of collagen synthesis (miR-29b)	(113,114)
miR-150 and miR-21	Decreased	Heart	Decrease of the levels of miRNAs in case of AF	In atrial fibrillation, miR-21 promotes structural remodelling by driving atrial fibrosis through fibroblast activation, while miR-150 appears to exert a protective role by regulating inflammatory signalling pathways associated with atrial remodelling and disease progression	(115,252)
miR-126, miR-17, miR-92a, and the inflammation-associated miR-155	Decreased	Heart	Circulating levels of vascular and inflammation-associated miRNAs that were significantly downregulated in patients with coronary artery disease	miR-126, miR-17, and miR-92a are endothelial-enriched miRNAs that regulate vascular integrity and angiogenesis, while miR-155 is derived from immune cells and modulates inflammatory responses; their downregulation in CAD reflects impaired endothelial function and chronic vascular inflammation	(116)
miR-375	Increased	T1D	Elevated as a biomarker of pancreatic beta cell death	miR-375 regulates pancreatic islet development and β-cell mass by modulating insulin secretion and cellular survival pathways, and its release during β-cell apoptosis reflects its active involvement in β-cell maintenance and turnover	(118)
miR-326	Increased	T1D	Elevated in peripheral blood lymphocytes of T1D patients with ongoing islet autoimmunity	miR-326 contributes to type 1 diabetes autoimmunity by promoting Th17 cell differentiation and potentially impairing immune regulation through suppression of Ets-1 and the vitamin D receptor, both of which are involved in modulating T-cell responses	(121)
miR-21a and miR-93	Decreased	T1D	Downregulated in peripheral blood mononuclear cells from T1D patients	miR-21a and miR-93 contribute to immune dysregulation in T1D by modulating pro-apoptotic and inflammatory signalling in peripheral blood mononuclear cells, with their downregulation potentially enhancing resistance to apoptosis and sustaining chronic inflammation	(120)
miR-152, miR-30a-5p, miR-181a, miR-24, miR-148a, miR-210, miR-27a, miR-29a, miR-26a, miR-27b, miR-25, and miR-200a	Increased	T1D	Clinically elevated in serum of patients with T1D	miR-152, miR-30a-5p, miR-181a, miR-24, miR-148a, miR-210, miR-27a, miR-29a, miR-26a, miR-27b, miR-25, and miR-200a are implicated in apoptosis regulation and β-cell network maintenance, suggesting their collective role in pancreatic islet stress responses and early pathophysiological remodelling during T1D onset	(122)
miR-23a and miR-126	Increased	T2D	Reliable biomarkers for the early detection of T2D	miR-23a and miR-126 regulate key metabolic and vascular pathways in T2D, with miR-23a implicated in insulin signalling and glucose transport via SMAD4 modulation, and miR-126 supporting endothelial repair and angiogenesis by enhancing VEGF signalling through suppression of SPRED1 and PIK3R2	(123,124)
miR-140-5p	Decreased	Osteoarthritis	Expressed in cartilages and chondrocytes. Its expression gets downregulated due to aging	miR-140-5p plays a protective role in cartilage by regulating chondrocyte senescence, inflammation, and extracellular matrix homeostasis, and its downregulation in osteoarthritis contributes to cartilage degradation through increased expression of catabolic and pro-inflammatory mediators	(253)
miR-486	Decreased	Sarcopenia	This miRNA is involved in skeletal muscle cells proliferation providing the muscle mass.	miR-486 supports skeletal muscle maintenance by promoting myoblast differentiation and muscle protein synthesis via the PI3K/AKT pathway, and its downregulation in sarcopenia contributes to muscle mass loss and impaired proteostasis	(106)
miR-146a	Decreased	Sarcopenia	Downregulation of miR146a could accelerate the aging process, resulting in poor muscle strength noted in sarcopenic patients	miR-146a regulates skeletal muscle strength by suppressing inflammation and oxidative stress through targeting TRAF6 and IRAK1, and its downregulation in sarcopenia contributes to impaired redox balance and muscle function decline	(106)
miR-101-3p and miR-142-5p	Decreased	Frailty	Downregulated in patients with frailty	miR-101-3p and miR-142-5p contribute to frailty pathophysiology by regulating key processes such as cellular senescence, oxidative stress response, immune signalling, and neuronal maintenance, with their downregulation disrupting homeostatic mechanisms essential for resilience in ageing	(110)

The essential function of miRNAs in the cardiovascular system is confirmed by the discovery that the absence of the miRNA-processing enzyme DICER results in abnormal angiogenesis and the embryogenesis of the heart. Furthermore, endothelial cells can express several miRNAs that either promote or suppress angiogenesis. miRNAs linked to vascular function and inflammation are significantly decreased in individuals with coronary artery disease [116].

Modifying how miRNAs are expressed can disrupt glucose and lipid metabolism, manifesting as metabolic disorders [117]. For example, miRNAs regulate the formation and differentiation of β-cells and the release of insulin and its effects on target tissues. MiRNAs also directly regulate genes responsible for β-cell survival and insulin exocytosis. Levels of miR-375 in the bloodstream of non-obese diabetic (NOD) mice can serve as a biomarker for the death of β-cells, and their level can be considerably elevated two weeks before the onset of Type 1 Diabetes Mellitus (T1DM) [118]. The main mechanism by which they contribute to the development of both Type 2 Diabetes Mellitus (T2DM) is through the development of insulin resistance [119]. Elevated and downregulated levels of certain miRNAs involved in regulating autoimmune processes have been observed in the peripheral blood lymphocytes and monocytes of patients with T1DM [120,121]. A study by Nielsen et al. demonstrated the increased expression of twelve serum miRNAs in individuals diagnosed with T1DM [122]. In T2DM, serum miR-23a and miR-126 have been proposed as dependable biomarkers for the early identification of T2D [123,124].

### 3.3 Alzheimer's disease

Downregulation in hsa-miR-181c-5p and hsa-miR-29c-3p has been shown in both brain and blood samples of AD patients, while other markers were upregulated in brain and downregulated in blood, including hsa-miR- 125b-5p, hsa-miR-146a-5p, and hsa-miR-223-3p [125]. To explain this, if a disease-related biomarker is increased in a tissue-specific concentration, only a fraction of it is leaked into circulation, or the rising levels of the biomarker in tissue-specific concentration may be counterbalanced by decreased cellular miRNA secretion [126]. The microRNA let-7b is increased in the cerebrospinal fluid (CSF) of AD patients. Extracellular let-7 can activate neuronal the RNA-sensing toll-like receptor (TLR)-7, contributing to neurodegeneration. Indeed, wild-type mice injected intrathecally with let-7b demonstrated marked axonal injury and neuronal loss [127]. Moreover, increased expression of miR-34c, miR-30a, and miR-27a as part of a six-ncRNA signature in CSF has been used to predict and diagnose AD dementia [128]. miR-34c expression was also increased in the hippocampus of mouse models exhibiting amyloid deposition and memory decline associated with ageing and in postmortem brain specimens of AD patients [129,130]. Another study has reported lower expression levels of miR-92a-3p and miR-486-5p and relatively higher levels of miR-29a-3p observed in AD patients’ plasma [131]. These biomarkers could play a role in synaptic transmission, structural functions, cell signalling and metabolism [132,133].

From a therapeutic standpoint, miRNA-132 has demonstrated neuroprotective effects but is significantly downregulated in the brains of AD patients [134]. Supplementing AD mice with miR-132 can mitigate amyloid and Tau pathology and enhance memory function [[135], [136], [137], [138], [139]]. Nonetheless, how changing miR-132 expression in the brain affects biological cascades and molecular networks that span several cell types to protect against or precipitate neurodegenerative changes remains investigational. Along similar lines, the downregulation of miR-195 has been linked to AD, potentially involving the β-site APP cleaving enzyme 1 (BACE1) protein and amyloid precursor protein (APP) aimed at Aβ deposition [140]. Hence, studies have attempted to use liposomal microRNA-195 as a possible therapy for AD: intravenous nanoliposomes encapsulating [59,60] (PEI)/miR-195 complex (DPMT@PEI/miR-195) can alleviate cognitive decline in APP/PS1 mice—an animal model of AD) [141].

MicroRNA-485-3p was shown to be overexpressed in the brain tissues and CSF of AD patients. A study conducted on a transgenic mouse model of AD reported that the antisense oligonucleotide (ASO) of microRNA-485-3p decreased the development of tau pathology, Aβ plaque accumulation, neuroinflammation, and cognitive decline [142]. MiR-485-3p ASO increased Aβ clearance through CD36-mediated phagocytosis of Aβ both *in vitro* and *in vivo* [142]. Additionally, miR-485-3p ASO lowered neuronal apoptosis, reducing the levels of truncated tau, and lowered levels of pro-inflammatory cytokines such as TNF-α and IL-1β [142]. A recent study by Ge et al. showed that plasma levels of miR-431 were decreased in patients with mild cognitive impairment and AD [143]. In APP/PS1 mouse models of AD, miR-431 overexpression in the hippocampus CA1 area improved synaptic plasticity and attenuated memory deficits whilst not affecting Aβ levels. Mechanistically, this effect was mediated by suppression of Smad4 mRNA by miR-431, as evidenced by Smad4 overexpression reversed the apparent benefits of miR-431. Hence, multiple signalling pathways regulated by miRNAs appear to be favourable targets for developing therapies against AD.

### 3.4 Osteoporosis and osteoarthritis

A case-control study compared circulating miRNA profiles of 36 patients with fragility fractures against 39 controls to identify biomarkers for fracture risk [144]. Significant differences were found across both groups, with 46, 52, and 55 miRNAs differentially expressed in premenopausal women, postmenopausal women, and male subjects, respectively. Importantly, 19 miRNAs were consistently differentially expressed across all subgroups. Eight miRNAs (miR-152-3p/-30e-5p/-140-5p/-324-3p/-19b-3/-335-5p/-19a-3p and -550a-3p) demonstrated an excellent correlation with an area under the curve (AUC) > 0.9 [144]. Various other clinical studies have profiled miRNAs in the blood of osteoporosis and osteoarthritis patients to identify differentially associated miRs. Still, there is little agreement between studies due to a large degree of heterogeneity, little standardization, and a high risk of bias in most studies. Nevertheless, a systematic review demonstrated the upregulation of certain miRs is consistently associated with osteoporosis (miR-125b, miR-100, miR-148a, and miR-24) and osteoarthritis (miR-146a, miR-155, and miR-98) [145]. These findings also suggest that miRNAs play a role in modulating the biology of osteoporosis and osteoarthritis.

Mechanistically, miRNAs regulate various signalling pathways, including RANKL/RANK/OPG, MAPK, and PI3K/AKT signalling pathways, that play a role in regulating the differentiation, proliferation, resorption, and apoptosis of osteoclasts [146,147]. miR-143-3P in osteoblasts is associated with the decrease in OPG and increase in RANKL, which in turn stimulates the formation of osteoclasts [148]. MiR-140-5p and miR-140-3p are expressed in chondrocytes. A decrease in the former is seen in aged mice compared to young and healthy mice, suggesting its role as a contributor to the development of osteoporosis [[149], [150]]. Saferding et al. identified miR-146a as a critical regulator of age-related bone loss. Specifically, its downregulation increases bone mass by enhancing osteoblastic activity through activation of Wnt1 and Wnt5a signalling [151]. Indeed, miR-146a-deficient mice were protected from ovariectomy-induced bone loss. In humans, elevated miR-14a correlated with fragility fractures [151]. These findings highlight the role of miR-146a in the pathogenesis of age-related osteoporosis and its utility as a biomarker and potential as a therapeutic target. Lastly, the expression of miR-19a-3p decreases with age in posterior iliac crest biopsy samples in older versus younger women, accompanied in mice with an increase in cellular senescence markers (p16^*ink4a*^ and p21^*cip1/waf1*^). This effect was reversible with miR-19a-3p overexpression [76].

Osteoarthritis is defined as degeneration of the articular cartilage and is a common age-related disease. Lin et al. demonstrated that miR-653-5p was downregulated in cartilage tissues and chondrocytes derived from osteoarthritis patients [152]. Further functional validation studies demonstrated that miR-653-5p suppressed chondrocyte senescence by repressing the IL-6/JAK/STAT3 signalling pathway. Furthermore, exogenous administration of agomiR-653-5p significantly attenuated chondrocyte senescence and cartilage destruction [152]. Along similar lines, the downregulation of miR-29b-5p in chondrocytes and articular cartilage with age has been associated with osteoarthritis and biologically to the upregulation of senescence markers (p16^*ink4a*^ and p21^*cip1/waf1*^) as well as matrix metalloproteinases, which have an established role in osteoarthritis pathogenesis [153]. Exogenously administering agomiR-29b-5p conjugated to a stem cell-recruiting peptide SKPPGTSS led to successful stem cell homing, differentiation into chondrocytes, and cartilage regeneration [153].

### 3.5 Cancer

As miRNAs play key roles in most cellular processes, it is plausible that any dysregulation in their function could affect one or more of the carcinogenic hallmarks like replicative immortality, sustained proliferative signalling, metastasis, etc. [154]. For instance, miR-1269 is overexpressed in gastric cancer and inhibits the expression of RASSF9, a tumour suppressor that inhibits cell cycle progression [155]. On the other hand, miR-9 can decrease the expression of the CDK6-cyclin D1 complex that stimulates cell cycle progression; hence, its under-expression would likely contribute to the proliferation of tumorigenic OSCC cells [156]. A study demonstrated that miR-29b targeted insulin-like growth factor 1 (IGF1), a key player in the PI3K/Akt signalling pathway implicated in CRC tumorigenesis. miR-29b also had a positive regulatory effect on IRF1, which, in turn, stimulates miR-29b expression, creating a positive feedback loop.

Certain miRNAs are crucial for protective cellular responses to external stressors, including hypoxia, oxidative stress, and DNA damage. These miRNAs have tumour-suppressive roles, and their aberrant under-expression or loss-of-function leads to abnormalities that contribute to tumorigenesis, such as enhanced cell growth and invasion, increased resistance apoptosis, and decreased sensitivity to cytotoxic therapies, all of which feature as hallmarks of carcinogenesis [157]. Based on their inhibition of tumour-suppressive messenger RNAs, other miRNAs can become “oncomiRs” in cancer development following any aberrant overexpression or gain of function [158]. Table 4 summarizes the role of different miRNAs in various cancer types**.**

**Table 4:** Association between miRNAs and different cancer types.

Cancer Types	Related miRNA(s)	Association with tumour	Upregulated or downregulated	Mechanism of Action	References
Squamous Cell Carcinoma	miR-9	Tumour Suppressive	Down	miR-9 exerts tumour-suppressive effects in squamous cell carcinoma by directly targeting CDK6 and Cyclin D1, leading to G1/S cell cycle arrest, reduced proliferation, and enhanced apoptosis.	(156)
Hepatocellular Carcinoma	miR-145	Tumour Suppressive	Down	miR-145 functions as a tumour suppressor in hepatocellular carcinoma by directly targeting and downregulating ADAM17, which in turn reduces MMP-9 expression, thereby inhibiting cancer cell proliferation and tumour progression through suppression of the ADAM17/MMP-9 signalling axis.	(160)
Gastric Cancer	miR-1269	Oncogenic	Up	miR-1269 promotes gastric cancer progression by directly targeting the tumour suppressor RASSF9, thereby activating the AKT and Bcl-2/Bax signalling pathways to enhance cell proliferation, drive G1/S cell cycle transition, and inhibit apoptosis.	(155)
Hepatocellular Carcinoma	miR-487a miR-21-5	Oncogenic	Up	miR-21-5p promotes NASH-associated hepatocarcinogenesis by suppressing PPARα, thereby enhancing lipotoxicity, inflammation, and oncogenic signalling, while miR-487a facilitates HCC proliferation and metastasis by targeting PIK3R1 and SPRED2, activating AKT and MAPK pathways respectively.	(162,164)
All Cancer Types	miR-9c miR-79	Oncogenic	Up	miR-9c and miR-79 promote tumour progression by directly targeting and downregulating the ETS-family tumour suppressor Pnt, thereby abrogating Ras-induced cellular senescence and facilitating malignancy in Ras-activated tumors.	(163)
Colorectal Cancer (CRC)	miR-29b	Tumor Suppressive	Down	miR-29b functions as a tumour suppressor in colorectal cancer by directly targeting IGF1, inhibiting the PI3K/Akt signalling pathway, reducing cell proliferation and migration, and promoting apoptosis.	(254)
Pheochromocytomas or paragangliomas (PPGLs)	miR-210	Tumor Suppressive	Down	miR-210 modulates hypoxia-driven tumour behavior in PPGLs by regulating genes involved in mitochondrial metabolism, cell cycle control, and DNA repair, and its dysregulation may influence malignant transformation through impaired cellular adaptation to oxidative stress.	(255)
Colorectal cancer (CRC)	miR-103a-3p, miR-127-3p, miR-151a-5p, miR-17-5p, miR-181a-5p, miR-18a-5p and miR-18b-5p	–	Up	miR-103a-3p, miR-127-3p, miR-151a-5p, miR-17-5p, miR-181a-5p, miR-18a-5p, and miR-18b-5p contribute to colorectal cancer progression by regulating key oncogenic pathways including p53, PI3K/AKT, and KRAS signalling, enhancing tumour cell proliferation, invasion, DNA damage evasion, and metastatic potential.	(256)
Cervical cancer	The ratio between miR-25/92a and miR-22/29a groups	Oncogenic	Up	The elevated miR-25/92a to miR-22/29a ratio in cervical cancer reflects enhanced oncogenic signalling driven by HPV E6/E7 oncoproteins, which upregulate miR-25 and miR-92a via p53 and E2F1 deregulation, promoting proliferation and inhibiting apoptosis, while suppressing tumor-suppressive miR-22 and miR-29a pathways involved in cell cycle control and differentiation.	(257)
Gastric cancer	miR-491-5p	Tumor Suppressive	Down	miR-491-5p acts as a tumour suppressor in gastric cancer by directly targeting the histone demethylase JMJD2B, thereby inhibiting cancer cell proliferation, migration, and invasion through epigenetic repression of oncogenic pathways.	(258)
Invasive breast cancer	Exosomal miR-223-3p	Oncogenic	Up	Exosomal miR-223-3p promotes invasive breast cancer progression by downregulating the tumour suppressor EPB41L3, thereby enhancing cancer cell proliferation, invasion, and malignant transformation.	(259)
Glioma	miR-21, miR-222 and miR-124-3p	–	Up	miR-21, miR-222, and miR-124-3p are upregulated in glioma and contribute to tumor progression by promoting cell proliferation, invasion, and anti-apoptotic signaling, with miR-21 and miR-222 modulating oncogenic targets like PTEN and p27, and miR-124-3p influencing glioma-specific regulatory networks such as MGMT expression.	(260)
Breast cancer	miR-21	–	Up	miR-21 promotes breast cancer progression by directly targeting tumour suppressor genes such as PTEN and PDCD4, inhibiting apoptosis and enhancing cell proliferation, migration, and invasion.	(261)
Lung cancer	miR-33a-5p and miR-128-3p	Tumor Suppressive	Down	miR-33a-5p and miR-128-3p act as tumour suppressors in lung cancer by inhibiting proliferation, epithelial-mesenchymal transition (EMT), and metastasis through direct targeting of oncogenic regulators such as CAND1 and PFKL, respectively, and by modulating pathways involved in cell cycle progression, glycolysis, and metastatic signalling.	(262)
Prostate cancer	miR-214	Tumor Suppressive	Down	miR-214 functions as a tumour suppressor in prostate cancer by directly targeting and downregulating PTK6, thereby inhibiting cell proliferation, migration, invasion, and epithelial–mesenchymal transition (EMT), while also inducing apoptosis and sensitizing cells to chemotherapeutic agents.	(263)
Head and neck squamous cell carcinoma (HNSCC)	mgmiR9-1, mgmiR124-1, mgmiR124-2, mgmiR124-3, mgmiR129-2, mgmiR137, and mgmiR148a	–	Up	In HNSCC, hypermethylation-mediated silencing of tumour-suppressive miRNAs—miR-9-1, miR-124-1/2/3, miR-129-2, miR-137, and miR-148a—disrupts post-transcriptional regulation of oncogenes involved in cell cycle progression, proliferation, and migration, thereby promoting malignant transformation and tumour development.	(264)
Primary central nervous system lymphoma (PCNSL)	miR-21, miR-19b, and miR-92a	–	Up	In PCNSL, miR-21, miR-19b, and miR-92a are upregulated and contribute to lymphoma pathogenesis by acting as oncogenic regulators—miR-21 suppresses tumour suppressors like PTEN, while miR-19b and miR-92a, members of the oncogenic miR-17∼92 cluster, promote proliferation and survival through activation of PI3K/AKT and anti-apoptotic signalling pathways.	(265)
Synovial sarcoma (SS)	miR-92b-3p	Secreted by tumour	Up	miR-92b-3p is actively secreted by synovial sarcoma cells via tumour-derived exosomes and may contribute to tumour progression through intercellular communication, while its circulating levels serve as a proxy for tumour burden and dynamic disease status.	(266)
Esophageal squamous cell carcinoma (ESCC)	miR-1	Tumor Suppressive	Down	miR-1 acts as a tumour suppressor in ESCC by directly targeting and downregulating fibronectin 1 (FN1), thereby inhibiting cell proliferation, migration, and invasion, while promoting apoptosis through disruption of extracellular matrix signalling pathways essential for tumour progression.	(267)
Advanced cervical cancer	miR-944	Oncogenic	Up	miR-944 promotes cervical cancer progression by targeting tumour suppressor genes such as HECW2 and S100PBP, facilitating cell proliferation, migration, and invasion, with its upregulation strongly associated with HPV E6/E7 expression, larger tumour size, lymph node metastasis, and poor overall survival.	(268)
Oral squamous cell carcinoma (OSCC)	miR-1290	Oncogenic	Down	miR-1290 promotes oncogenic processes in OSCC by targeting tumour suppressor genes such as FOXC1, GLIPR1, and hMSH2, facilitating epithelial–mesenchymal transition, chemoradiotherapy resistance, and cancer stem cell-like phenotypes, with its dysregulation linked to poor tumour differentiation and treatment outcomes.	(269)
Colorectal cancer (CRC)	Circ_0026344 (a miRNA sponge for miR-21 and miR-31)	Tumor Suppressive	Down	circ_0026344 suppresses colorectal cancer progression by acting as a competitive endogenous RNA (ceRNA) that sponges oncogenic miR-21 and miR-31, thereby restoring the expression of tumour suppressor targets and inhibiting CRC cell proliferation, invasion, and promoting apoptosis.	(270)
Pancreatic ductal adenocarcinoma (PDAC)	miR-296-5p	Oncogenic	Up	miR-296-5p acts as an oncogenic microRNA in PDAC by directly targeting the pro-apoptotic gene BOK, thereby promoting epithelial–mesenchymal transition (EMT), enhancing cell invasion, and inducing chemoresistance through inhibition of apoptosis.	(271)
Small intestinal neuroendocrine tumors	miR-375	Oncogenic	Up	miR-375 is highly upregulated in small intestinal neuroendocrine tumors and promotes tumor progression by regulating Wnt and Hippo signaling pathways, contributing to neuroendocrine lineage differentiation and serving as a biomarker of poor prognosis when downregulated in metastatic lesions.	(272)
TNM stage II and III colon cancer	A 16-miRNA signature including miR-143-5p, miR-27a-3p, miR-31-5p, miR-181a-5p, miR-30b-5p, miR-30d-5p, miR-146a-5p, miR-23a-3p, miR-150-5p, miR-210-3p, miR-25-3p, miR-196a-5p, miR-148a-3p, miR-222-3p, miR-30c-5p and miR-223-3p	Oncogenic (except miR-25)	Up	The 16-miRNA signature—including oncogenic miRNAs such as miR-31-5p, miR-181a-5p, miR-27a-3p, and others—promotes colon cancer progression through coordinated regulation of key oncogenic pathways like RAS-MAPK and PI3K-AKT, while miR-25-3p, uniquely downregulated, likely acts as a tumour suppressor by targeting Smad7 to inhibit TGF-β signalling.	(273)
Ovarian cancer	miR-135a-3p	Tumor Suppressive	Down	miR-135a-3p functions as a tumour suppressor in ovarian cancer by downregulating oncogenic targets such as BIRC3, GABRA3, and SPANXB1/2, leading to reduced cell proliferation, increased chemosensitivity to cisplatin and paclitaxel, and suppression of xenograft tumor growth.	(274)
Chronic lymphocytic leukemia (CLL)	miR-155-5p	Oncogenic	Up	miR-155-5p acts as an oncogenic driver in CLL by targeting key regulators such as FOXO3 and HIF1A, thereby disrupting cell cycle control, promoting leukemic B-cell survival, and conferring resistance to apoptosis, while its overexpression independently predicts poor overall survival regardless of IGHV status or CD38 expression.	(275)
Prostate cancer	miR-424-3p	Tumor suppressive	Down	miR-424-3p functions as a tumour suppressor in prostate cancer by modulating immune checkpoint pathways, specifically downregulating CTLA-4 and PD-L1, thereby enhancing antitumor immune responses and reducing tumour aggressiveness, with its low expression significantly associated with poor clinical outcomes and advanced pathological features.	(276)
Breast cancer	miR-362-3p	Tumor suppressive	Down	miR-362-3p exerts tumour-suppressive effects in breast cancer by directly targeting the 3′UTR of the oncogenic hERG (KCNH2) potassium channel, leading to reduced hERG expression and current, inhibition of cell proliferation, and G0/G1 cell cycle arrest.	(277)
Clear cell renal cell carcinoma (ccrcc)	miR-130b, miR-18a, and miR-223	Oncogenic	Up	miR-130b, miR-18a, and miR-223 promote ccRCC progression by regulating transcriptional and signalling pathways such as mTOR, FoxO, and endocytosis, contributing to tumour proliferation, migration, and poor prognosis, with their combined upregulation forming a robust miRNA signature predictive of patient survival.	(278)
Breast cancer	miR-196a	Oncogenic	Up	miR-196a promotes breast cancer progression and therapeutic resistance by targeting tumour suppressor genes such as HOXA5, ANXA1, and CDKN1B (p^27^K^ip1^), enhancing proliferation, inhibiting apoptosis, and facilitating epithelial-to-mesenchymal transition through estrogen receptor alpha (ERα)-driven transcriptional activation.	(279)

Tumor-suppressive miRNAs can be under-expressed in neoplastic conditions. For instance, oral squamous cell carcinoma (OSCC), the most common type of oral cancer, remains to be one of the leading causes of death worldwide. Due to it being asymptomatic at an early stage, OSCC is often asymptomatic at early stages and, therefore, is typically diagnosed at an advanced stage, portending a poor prognosis. Sun et al. reported that miR-9 was downregulated in OSCC patients [159], which may reduce cell proliferation and prevent cell migration when overexpressed [156]. There is also evidence suggesting that miRNAs can be utilized to suppress certain aspects of carcinogenesis. For instance, in cases of hepatocellular carcinoma (HCC), miR-145 was found to inhibit ADAM17 (A disintegrin and metalloproteinase 17), consequently inhibiting cell proliferation and growth in liver cancer cells [160].

More than half of known miRNA genes are found in fragile locations or genomic areas linked with cancer, indicating that miRNAs may be more important than previously believed to the pathophysiology of a small number of human malignancies. Overexpressed miRNAs may act as oncogenes and stimulate cancer growth by inhibiting genes involved in tumour suppression, cell differentiation, and/or apoptosis [161]. In this context, miR-1269 upregulated promoted cell proliferation and cell cycle G1-S transition and suppressed apoptosis. Conversely, inhibition of miR-1269 had the reverse effects [155]. In another study, 198 patients with HCC—a highly metastatic cancer—were recruited to evaluate the association between levels of miR-487a and clinicopathological features and prognosis of the cancer. High levels of serum miR-487a corresponded to more metastases—via SPRED2-induced mitogen-activated protein kinase signaling—and invasion— via PIK3R1-mediated AKT signalling—both the *in vivo* and *in vitro*, associated with poor prognosis [162].

MiRNAs may also contribute to tumorigenesis by inhibiting cellular senescence, which represents a critical physiologic tumour-suppressive mechanism induced by strong mitogenic stimuli such as the activation of oncogenes or inactivation of tumour-suppressor genes. The effect of cancer therapies such as chemoradiation is mediated by the induction of apoptosis or senescence in tumour cells, leading to tumour shrinkage and growth arrest, respectively. The activation of Ras signalling is one of the earliest steps of oncogene-driven tumour cell proliferation. Yet, the Ras signalling pathway is also a potent activator of the process of cellular senescence. A transcriptional activator called Pointed (Ptd) is a critical step in cellular senescence induction, so any dysfunction or inhibition in this protein represses senescence and promotes tumour development. A loss of cell polarity and structural organization triggers a cascade of events that eventually release miRNA-9c and miRNA-79, both of which are inhibitors of Ptn and, thus, oncogenic [163].

MiRNAs may impact the sensitivity of cancer cells to therapy. Patients with cisplatin-resistant HCC exhibit higher levels of miR-21-5p as compared to notably higher than others with the cancer. miR-21-5p suppressed the expression of FASL, attenuating cisplatin-induced apoptosis [164].

While considerable strides have been taken to predict and elucidate the specific pathways in which miRNAs are involved in the past years, these predictions require experimental validity for clinical applications. The loss-of-function approach is the best experimental approach to analyze miRNA-associated pathways. Using miRNA sponges represent is one such method [165]. Sponge RNAs contain complementary binding sites to a family of miRNAs, allowing them to inhibit the function of structurally similar miRNAs [165]. MiRNA sponges also have considerable applications as therapies in preclinical models, as they may block the activity of oncogenic miRNAs. For example, the Notch pathway in lung adenocarcinoma regulates cell differentiation, proliferation, survival, and angiogenesis and may be critically involved in cancer progression [166]. The tumour-suppressor protein Numb promotes ubiquitin-mediated degradation of membrane-bound Notch1 receptors [167,168]. miR-146, however, silences Numb expression, indirectly activating the Notch pathway. Administration of TUSC-7, a miRNA sponge, in lung adenocarcinoma blunted miR-146-mediated degradation of Numb, reducing the self-renewal of adenocarcinoma stem cells.

Other sponges may contribute to tumorigenesis and metastasis, such as FAM225A in nasopharyngeal carcinoma (NPC). FAM225A blocks the activity of miR-590-3p and miR-1275, leading to increased levels of integrin β3 (ITGB3) and, consequently, activation of the FAK/PI3K/Akt signalling pathway to promote NPC cell proliferation and invasion [169]. Similarly, circMMP9, a circular RNA sponge, has been found to target miR-124 in glioblastoma multiforme (GBM) cases. In patients with GBM, miR-124 can be significantly downregulated, leading to increased cell proliferation, whereas exogenous transfection of miR-124 into GBM cells induces cell cycle arrest [170]. Due to the protective functions of miR-124, increased expression of circMMP9 accelerates GCM cell proliferation, cell migration and invasion, and tumorigenesis.

Breast Cancer (BC) is the most common cancer diagnosed in women [171], with a very complex pathogenesis involving various genetic, hormonal, environmental, and lifestyle factors. As an example, Table 5 summarizes the role of miRNA sponges in the pathogenesis of BC.

**Table 5:** miRNA sponges in breast cancer.

Sponge	Action	Upregulated/downregulated	miRNA Target	Reference
circRNF20	Promoting the proliferation and Warburg Effect of breast cancer cells	Up	miR-487a	(280)
circCDYL	Promoting autophagy, enhancing malignant progression	Up	MiR-1275-ATG7	(281)
CircRNA_0025202	Tamoxifen-Sensitivity	Down	MiR-182-5p	(282)
MiRNA-10b sponge	Inhibited miR-10b and upregulated HOXD-10	(Synthetic)	MiRNA-10	(283)
CircNR3C2	Promotes tumour-suppressive effects of HRD1	Down	MiR-513a-3p	(284)
SNHG3	Positively regulate PKM expression, inhibit mitochondrial oxidative phosphorylation, enhance BC cell proliferation	Up	MiR-330-5p	(285)
CircVAPA	Promote migration and invasion capacity of BC	Up	MiR-130a-5p	(286)
Circ_0052112	Cell migration and invasion in BC	Up	MiR-125a-5p	(287)
LINC00467	Proliferation, migration, invasion, and epithelial-to-mesenchymal transition (EMT) of BC cells	Up	MiR-138-5p	(288)
MALAT1	Cellular migration and proliferation of BC cells	Up (under hypoxia)	MiR-3064-5p	(289)
CircZNF609	Elevate p70S6K1 expression and contribute to BC progression	Up	MiR-145-5p	(290)
Circ_ZFR	Proliferation, migration, invasion, EMT	Up	MiR-223-3p	(291)
CircAAGAB	Reduced cell colony formation, cell migration, increased radio sensitivity	Down	MiR-378 h	(292)
CircUBR5	Sustaining malignant growth and metastasis of triple-negative breast cancer (TNBC) cells	Up	MiR-1179	(293)
GAS5	Promoting apoptosis and inhibiting cell proliferation	Down	miR-21-5p, miR-221-3p, miR-196a-5p, miR-378-5p, miR-216	(294)
CircGFRA1	Proliferation and inhibition of apoptosis	Up	MiR-34a	(295)
CircUBE2D2	Lymph node metastasis and adverse prognosis, doxorubicin resistance	Up	MiR-512-3p	(296)
NDRG1-OT1	Promote tumour growth and migration	Up (under Hypoxia)	MiR-875-3p	(297)
PPP1R14B-AS1	Oncogenicity of breast cancer	Up	MiR-134-3p	(298)

## 4 Conclusions and perspectives

We have detailed numerous studies showing that miRNAs influence the biology of ageing and a variety of age-related diseases. This, however, is no guarantee of their clinical value. Looking through the lens of patients and doctors means prioritizing the translation of those therapies or disease markers that serve the ultimate goal of making patients live longer or live better. For these promising biological advances to be actualized into patient benefit, a painstaking process of clinical translation must be undertaken. Concerning the potential of miRNAs as disease/ageing biomarkers, their ability to be correlated with certain aspects of the disease in observational studies is not sufficient evidence to justify their use in clinical practice. Their profile must correlate with a change in clinical state in a manner that is meaningful to patients (i.e., predicts their survival and/or health-related quality of life). In this sense, miRNAs would constitute a surrogate for clinical outcomes, and there is a rich history in medicine of surrogate outcomes that do not correlate with the endpoint that matters (e.g., HDL-C not improving mortality). The gold standard for establishing a robust correlation is a clinical trial-level validation, where miRNA profiles are monitored in a randomized cohort being treated for an age-related, chronic condition, and a correlation is made between their trajectory and clinical outcomes. Similarly, regarding therapy, strategies targeting miRNAs must be tried against the best available care in a setting that is adequately powered to detect changes in clinical endpoints. Though this will take years to achieve, much of the groundwork has already been laid by the promising preclinical studies detailed in this review.

## CRediT authorship contribution statement

**Rawad Turko:** Writing – review & editing, Writing – original draft, Validation, Methodology, Data curation, Conceptualization. **Amro Hajja:** Writing – review & editing, Writing – original draft, Project administration, Methodology, Data curation, Conceptualization. **Ahmad M. Magableh:** Writing – review & editing, Writing – original draft, Data curation. **Mohammed H. Omer:** Writing – review & editing, Writing – original draft, Project administration, Data curation. **Areez Shafqat:** Writing – review & editing, Writing – original draft, Project administration, Data curation, Conceptualization. **Mohammad Imran Khan:** Writing – review & editing, Writing – original draft, Data curation, Conceptualization. **Ahmed Yaqinuddin:** Writing – review & editing, Writing – original draft, Validation, Supervision, Project administration, Conceptualization.

## Declaration of competing interest

The authors declare that they have no known competing financial interests or personal relationships that could have appeared to influence the work reported in this paper.
